# Concordant physician-patient characteristics lose importance for Arab American women and their healthcare- cross-sectional study

**DOI:** 10.1016/j.lana.2022.100225

**Published:** 2022-03-03

**Authors:** Diane M Harper, Ananda Sen, Madiha Tariq, Christelle El Khoury, Elizabeth K. Haro, Emma Alman, Minal R. Patel, Ken Resnicow

**Affiliations:** a Department of Family Medicine, University of Michigan School of Medicine, Ann Arbor, 1018 Fuller Street, MI 48104, USA; b Department of Obstetrics & Gynecology, University of Michigan School of Medicine, Ann Arbor, MI, USA; c Department of Women’s Studies, College of Literature, Science and the Arts, University of Michigan, Ann Arbor, MI, USA; d School of Public Health, University of Michigan, Ann Arbor, MI, USA; e Arab Community Center for Economic and Social Services (ACCESS), Dearborn, MI, USA; f Department of Health Behavior & Health Education, University of Michigan School of Public Health, Ann Arbor, MI, USA; g Outreach and Health Disparities Research, University of Michigan Rogel Cancer Center, Ann Arbor, MI, USA; h Center for Health Communications Research, School of Public Health, University of Michigan, Ann Arbor, MI, USA

## Abstract

**Background:**

Arab American women have preferred women physicians of their own culture in the past. The primary aim of this study is to determine the current influence of religion/culture among MENA women and their preferences for physicians of same sex, culture, and religion on the avoidance and uncomfortableness of routine and women’s health exams.

**Methods:**

A cross sectional community survey including religiosity and the importance of physician matched sex, culture, and religion was completed. Outcome measures were avoidance of a routine physical exam, or a women’s health exam because of religious/cultural issues; and the uncomfortableness of the women’s health exam. Linear regression modeling was used to evaluate the association between outcomes and potential predictors, with significance assessed using a bootstrap method.

**Findings:**

The responses of 97 MENA women 30–65 years old showed that MENA women agreed that they would avoid routine health exams because of religious/cultural issues if their physician was of the same religion or culture as they were (*p* < 0.001, *p* < 0.05, respectively) or they had less education (*p* < 0.05). MENA women also avoided women’s health exams due to religious/cultural issues if her physician was of the same religion as she (*p* < 0.01).

**Interpretation:**

MENA women 30–65 years old may no longer be bound to a female physician of their same religion/culture for their health exams.

## Introduction

Arab American women from Middle East North African (MENA) heritage are not included in the national health surveillance studies as a separate race/ethnicity. Past qualitative reports have shared the reluctance of MENA women of the Muslim religion to have a genital/pelvic exam; and because of continued annual pressure from the primary care physician (PCP) to have a pelvic exam, they, therefore, do not attend visits for routine health care.^1^ Within the past decade, research focusing on the MENA women of Muslim religion showed a low rate of cervical cancer screening because, if disease was found, it, then, would be considered a punishment from God/Allah. A decade later, modesty issues had been addressed by healthcare systems for Muslim women, facilitating an uptake in cervical cancer screening, but still below other racial/ethnic groups.^2^ However, there are multiple religions, including several Christian denominations and Judaism, within the MENA community for whom the Muslim evidence may not represent the complete MENA experience. Others indicate that 77% of Arab American women received cervical cancer screening within three years,^3^ more than Hispanic women, but less than Black women.^4,5^ A community-based survey in 2019 reported 72% of MENA women were screened within the past three years but were unlikely to screen if they had been in the US for less than ten years or were single.^6^

The Arab American women in southeast Michigan are one of the fastest-growing population groups. There are increasing numbers of women who have spent less than ten years in the US.^7^ The primary aim of our work is to explore the association of MENA identity, religiosity, and the sociocultural preferences for same sex/religion/culture physician with the outcomes of agree/disagree with avoidance of routine healthcare exams and women’s health exams due to religious/cultural issues and agree/disagree that the women’s health exams are uncomfortable among American women of MENA descent in southeastern Michigan.

## Methods

### Survey design

The survey development is described in detail in previous publications.^8,9^ The survey was conducted at sites within the Arab American community in southeast Michigan or at their home, with or without an interviewer, in English or Arabic, and completed by paper, online, or by phone between May 1 and October 28, 2019.^10^ The survey was approved by the University of Michigan IRB (HUM00159558). All participants received an incentive for survey completion.

### Survey respondents

We restricted the analyses to MENA females 30–65 years of age. Adult routine health exams are recognized as a systematic way to solidify continuity relationships encouraging evidence-based screening, education on emerging health information, and a safe space to discuss sensitive issues, among others.^11^ Part of the routine health exam often includes cervical cancer screening: a woman’s health exam. The United States Preventative Services Task Force (USPSTF) recommends routine cervical cancer screening for women 30–65 years of age every 3–5 years in part because this range is the most important for screening and early detection of cervical cancer.^12–16^

### Ethics approval

Approval was obtained from the ethics committee of University of Michigan HUM00159558. The procedures used in this study adhere to the tenets of the Declaration of Helsinki.

### Consent to participate

Informed consent was obtained from all individual participants included in the study.

### Survey items

Our prior studies in this population informed the choice of the covariates that had been significant for predicting other cancer screenings for this study.^6,8,17^

#### Covariates.

Covariates assessed included age, marital status, education, annual income, occupation, and health insurance. Moreover, we included frequency of attendance at routine health exams and at routine Pap tests as surrogates for how perspectives on avoidance of exams were associated with actual attendance. Other covariates considered in this analysis included the woman’s parent’s country of origin, nativity, and length of time in the US. In addition, whether her physician was MENA and the importance of MENA to her identity on a scale from 0 to 10 (0 = not at all important; 10 = very important) were also included.

#### Measures of religiosity.

Several new items were generated or adapted for this study through interviews with community members and experts in MENA health.^18^ “*Religiosity*” is an overarching title for four specific measures. “*Religious denomination”* included Christian (Chaldean, Catholic, Assyrian, Coptic, Jehovah Witness), Jewish, Muslim, Baha’i, Druze or Alewi, Hindu, Buddhist, and Sikh; with optional text entry to include ten other self-described religions. “*Religious frequency of attendance*” was measured with a seven-item response scale to “How often do you attend religious services?”: never; less than once a year; about once or twice a year; several times a year; about once a month; 2–3 times a month; greater than 3 times a month. “*Religious salience*” was a four-item response scale to “How important is religion to you?”: very important; fairly important; fairly unimportant; not at all important. Finally, three other questions that measured the “*meaning of religion*” in one’s life which were developed from the multidimensional measurement of religiousness^19^: “God/Allah put me in this life for a purpose”; “God/Allah has a specific plan for my life”; “God/Allah has a reason for everything that happens to me” with the same four-item response scale: strongly disagree; disagree; agree; strongly agree.

#### Sociocultural preferences for the physician.

We examined three aspects of sociocultural preferences for healthcare providers using the same four-point response scale (1 = strongly disagree; 2 = somewhat disagree; 3 = somewhat agree; 4 = strongly agree). “It is important for my health care provider to be of the same sex as me, that is, a female doctor for a female patient”; “It is important for my health care provider to be of the same religion as me”; “It is important for my health care provider to be of the same culture as me.”

#### Survey outcomes.

Our outcomes included three questions assessing agreement/disagreement with avoidance of routine and women’s health care because of religious/cultural issues and with the uncomfortableness of the women’s health exam. Each question was scored on a 1–4 Likert scale of 1 = strongly disagree; 2 = somewhat disagree; 3 = somewhat agree; 4 = strongly agree. Two questions specifically linked religion/culture and the health care exam. “I have avoided getting a *routine physical* because of religious/cultural issues”; “I have avoided getting *women’s health exams (OBGYN*) because of religious/cultural issues”. The third question was based on prior significant pain reported among Arab Americans perceptions of Pap tests^20^: “I’m uncomfortable getting *women’s health exams (OBGYN*)”. We specifically left the women’s health exam terminology in both questions vague as it could include a range of exams such as breast exams, obstetrical care, sexually transmitted infection testing or cervical cancer screening. Likewise we left the interpretation of uncomfortable purposefully vague as it could include multiple dimensions of psychological, physical, and/or emotional discomfort.

### Statistical analysis

The means and standard deviation of each of the three outcome variables were calculated for each covariate, measure of religiosity, and sociocultural preferences for the physician. Each covariate is tested for significance for each of the outcomes under a univariate linear regression framework to identify associations that are significant at *p* < .10. Subsequently, a multivariable linear regression model was fit with the significant predictors only, to identify the most predictive covariate after accounting for multicollinearity. Although for a 4-point ordinal outcome, a natural choice is a proportional odds model, the proportionality assumption was violated for all models. On the other hand, the residuals from the linear regression demonstrated a departure from normality. Because of this, the association between the outcome and the covariates was evaluated based on the bootstrap resampling method that avoids any distributional assumption. Standard errors, 95% bias-corrected accelerated confidence intervals, and *p*-values were estimated based on one-thousand bootstrap samples. The software package was SPSS Statistics v 27.0.0 and Statistica v13.0.^21^

### Role of the funding source

The funding source was not involved in the study design; in the collection, analysis, and integration of data; in the writing of the report; and in the decision to submit the paper for publication.

## Results

The mean age of the 97 women was 43.6 years (SD 10.1) (Table 1). Over three-quarters of the women were married, and 40% had a high school education or less. More than half had incomes under $50,000 per year, and 52% were employed. All women had insurance, either private or public. 85% of women were born outside the United States with 27% being in the US for 10 years or less. 95% of women had parents of concordant ancestry with Iraq, Lebanon, Yemen, and Egypt being the most frequent countries. The majority population was split between Christian (38%) and Muslim (62%) religions. 64% had a PCP who was MENA, and her own MENA identity was very important to her (mean 8.0, SD 2.8). 33% of MENA women agreed that women’s health exams were uncomfortable; 24% avoided women’s health exams because of religious/cultural issues and 22% avoided routine health exams because of religious/cultural issues. The response rate to the questions about avoiding routine health exams was 93% (91/97), to avoiding women’s health exams was 95% (92/97) and being uncomfortable with women’s health exams at 93% (91/97). Health behaviors indicated that all women received a routine health exam but may have delayed it over time. In contrast, nearly one-fifth of women had never had a Pap test.

### Covariates/healthcare attendance

Univariate regression analyses indicate that only education was significantly associated with avoiding routine physicals: the less the MENA woman’s education, the more she avoided getting a routine physical because of religious/cultural issues (*p* < 0.05) (Supplemental Table 1). Education was carried forward to the multivariable regression for avoiding routine health exams due to religious/cultural issues.

### Measures of religiosity

The frequency of attendance at religious services was associated with avoidance of routine health exams. Specifically, the less frequent the attendance at religious services the greater the agreement that she avoided routine health exams because of religious/cultural issues (*p* < 0.05). None of the other measures of religiosity were associated with any of the outcomes (Table 2).

### Sociocultural preferences for the physician

Focusing on the attributes of the physician, the women’s preferences for the same sex, religion, and culture of her physician were associated in specific ways with our three outcomes (Table 3). The more strongly she agreed that the *sex* of her physician was important, the more she avoided getting a routine physical because of religious/cultural issues (*p* < 0.001) meaning that the more important it was to have a female physician, the more she avoided having a routine exam if the physician was not female. Likewise, the more strongly she agreed that the sex of her physician should be female, the more she agreed women’s health exams were uncomfortable (*p* = 0.002) indicating women’s health exams, in general, are seldom comfortable. Furthermore, the sex of her physician was only mildly associated with avoiding women’s health exams due to religious/cultural issues in univariate analysis (*p* = 0.014).

The importance of having her physician being of the same *religion* as she was, was significantly associated with all three outcomes as well. The more she agreed that a physician of the same religion was important, the more she agreed she avoided routine health exams because of religious/cultural issues (*p* < 0.001). Likewise, the more she agreed that having a physician of the same religion was important, the more she agreed she avoided getting women’s health exams because of religious/cultural issues (p=.003). Furthermore, the more she agreed that a physician of the same religion was important, the more she agreed she was uncomfortable with getting women’s health exams in general (*p* = 0.002).

Similarly, the importance of having her physician be of the same *culture* as she was, was also significantly associated with all three outcomes. The more important it was for the physician’s culture to match the woman, the more she agreed she avoided getting a routine physical because of religious/cultural issues (*p* < 0.001), avoided getting a women’s health exam (*p* = 0.02) and was uncomfortable getting a women’s health exam (*p* = 0.045).

### MENA identity

Neither the identity of the physician being MENA nor the importance of her MENA identity was associated with any outcome (Table 3).

### Predictors of routine and women’s health exams in multivariable models

The significant univariate analyses informed each of the three multivariable models (Tables 4–6). The model for avoiding routine health care because of religious/cultural issues included education as well as the frequency of religious service attendance and socio-cultural preferences for her physician (sex, religion, culture). Based on the bootstrap samples, the strongest association was demonstrated by the importance of having a physician of the same religion where respondents finding this to be of higher importance also had a greater tendency to avoid getting routine health exams because of religious/cultural issues (beta = 0.4, SE = 0.1, *p*-value < .001). Similarly, positive associations were found with the importance of having a physician of the same culture (beta = 0.212, SE = 0.09, *p*-value = 0.03) (Table 4). More simply stated, MENA women did not prefer a physician of their same religion or same culture for a routine health exam because of religious/cultural issues. Education had a significant negative association with the outcome as well. The more educated women tended to avoid routine health exams due to religious/cultural issues less than the less-educated women (beta = −0.083, SE = 0.04, *p*-value = 0.03).

In the multivariable analysis including all sociocultural factors, the only significant association with avoiding women’s health exams because of religious/cultural issues (Table 5) was the same religion physician preference (beta = 0.270, SE = 0.13, *p*-value = 0.03) (Table 5). Simply stated MENA women did not prefer a physician of their same religion for women’s health exams because of religious/cultural issues.

The preference for a physician of the same religion was statistically significant for predicting agreement with the uncomfortableness of women’s health exams (Table 6). A higher preference for a physician of the same sex or the same religion is associated with avoidance due to a higher level of discomfort with women’s health exams (beta = 0.232, SE = 0.11, *p*-value = 0.043) and (beta = 0.273, SE = 0.13, *p*-value = 0.037), respectively. Although preference for the same culture was borderline (*p* = 0.045) significant in univariate analysis, it lost significance in the multivariable model because it is highly collinear with preference for same religion (Spearman correlation = 0.64).

## Discussion

This is one of the largest surveys of Arab American women that incorporates religiosity and cultural identity applied to both the MENA woman and her PCP. In the past, concordance of religion/culture of her physician with the MENA women was a positive facilitator for any health exam.^5^ We are now reporting that this concordance has become a barrier to receiving routine and women’s health exams. We found that many risk factors previously identified for completing screening tests among MENA women were no longer significant.^5,6,8,22^ Routine health exams were no longer facilitated by having a same sex physician, despite the initial univariate analyses showing facilitation of healthcare by a religiously and/or culturally concordant physician. MENA women’s preferences have changed.

Parsing out the importance of religion among the MENA women we studied, our work immediately ruled out any significance of religious salience (the importance of religion to the MENA woman), religious denomination (Muslim vs other), or most religious meanings (the purpose/reason for her life directed by God/Allah) as predictors of any of the avoidance behaviors or uncomfortableness with women’s health exam outcomes. While the frequency of religious attendance initially was associated with routine health care exams because of religious/cultural issues, it did not persist in the multivariable model, indicating that the frequency of religious attendance was likely related to sociocultural networking rather than a monotheistic doctrine. This hypothesis is supported by Koenig’s work that links the belief in the transcendent (God, Allah, Vishnu, HaShem, Buddha, Dao or a Higher Power) to positive healthcare through the mediator of supportive community behaviors, not religion.^23,24^ The lack of significance of predictors that had been important in the past, shows the progression and evolution of the MENA women of our study with assimilating into the US healthcare system.^1,2,25^

Her own religion (Muslim/other) was not associated with the avoidance of *women’s* health exams or the *uncomfortableness* of the women’s health exams. However, the *religion of the physician* was significant for avoiding *women’s health* exams due to religious/cultural issues. MENA women who did not want their PCP to have their same religion agreed that they avoided getting *women’s* health exams because of religious/cultural issues. This is supported in other studies where MENA women feel they are being dismissively and negatively judged by their same religion PCP especially around sexual and reproductive health issues at a women’s health exam.^18,26^ Similarly, physicians of the same religion have been found to neglect to educate women about risks for sexually transmitted infections, contraception, menstrual hygiene, or other reproductive health issues, including cancer screening for which prevention is effective.^27–29^ Others attribute the lack of performing women’s health exams to the physician’s own feelings of incompetence with the exam or medico-legal fears of allegations of misconduct.^30^ Moreover, up to half of physicians feel their religious views are more important than the needs of their patients even to the point of not disclosing their bias to their patients.^31,32^ Moreover, physicians have been shown to be unable to ignore the influence of personal religious values when practicing medicine.^33^

In the final model predicting the uncomfortableness of getting women’s health exams, both having a same sex physician or a same religion physician modified the comfortableness of a women’s health exam, where the stronger the preference for sameness, the less uncomfortable the women’s health exam was considered. Women’s health exams engender feelings of embarrassment about undressing, worries of cleanliness and vaginal odor, and anxiety that the PCP may find something unusual about her sexual practices.^5,18,20,30,34^ These fears are usually mediated by having a female physician,^1,2,18,34^ as we saw in our study.

The *culture* of the physician was only significant in the final model predicting avoidance of routine physicals because of religious/cultural issues. Overall, it is not the religion, sex, or culture of the physician that creates the safe environment for a routine physical devoid of religious or cultural overtones, it is the relationship the woman has with her PCP.^17^ These insights are powerful observations of the evolution of immigrant cultures seeking necessary health care. Likewise, our insights show that physician behaviors of cultural competency and professional conduct are also necessary for MENA women to receive appropriate healthcare.

### Limitations

This is a cross-sectional convenience sample of MENA women in southeast Michigan, and as such, is subject to a possible sampling bias, meaning that these results may not be generalizable to all MENA women in the United States. This is apparent in the religious denominations of our study which do not reflect the distribution of Arab American religions throughout the United States (Roman Catholic (35%), followed by Muslim (24%), and then Eastern Orthodox Christian (18%).^7^ In addition, avoidance of exams and being uncomfortable with exams does not mean that the woman will not eventually accede to the health exams in her own time.^35^

Moreover, we restricted the survey sample to women 30–65 years of age because this was the age at which most preventive health care occurs, but this omits the women 18–29 years old who are at the highest risk for sexually transmitted diseases and in most need of contraception. The results of this study in a younger cohort may have different results.

## Conclusion

Current medical practices no longer support clinical breast exams, nor require a routine pelvic exam. Sexually transmitted diseases and cervical cancer testing can be performed with a urine sample or a self-swab for human papillomavirus (HPV), minimizing the need for invasive women’s health exams. Nevertheless, broadening physicians’ education about separating personal religious beliefs from women’s health care may be needed to change MENA women’s avoidance behaviors in seeking healthcare.

Sociocultural world events continue to challenge and change women’s knowledge, preferences, and actions about their healthcare. Our results highlight a research gap in the sensitive area of religion/ethics/medical practice/women’s rights and women’s healthcare. We have presented an updated view of the impact of religion and culture on the healthcare experiences of SE Michigan MENA women, which seems to have evolved from earlier research.

## Supplementary Material

1

## Figures and Tables

**Table 1:** Sociodemographic descriptors of respondents (*N* = 97).

	N	%
**Covariates**		
**Age, years (mean, SD)**	43.6	10.1
**Marital Status**		
Married	74	77.9
Single	21	22.1
**Education**		
High School or less	38	40.0
Some college	16	16.8
College	31	32.6
Post college	10	10.5
**Income**		
<$10K	14	15.6
$10-$49999	46	51.1
$50-$99999	19	21.1
>$100,000	11	12.2
**Occupation**		
Employed	44	51.8
Unemployed	21	24.7
Homemaker	18	21.2
Disabled	2	2.4
**Insurance**		
Private	29	40.8
Federal	42	59.2
None	0	0.0
**Born in US**		
Yes	15	15.5
No	82	84.5
**Length of time in US**		
10 years or less	20	27.4
More than 10 years	53	72.6
**Parent’s Country of Origin**		
Lebanon	30	30.9
Iraq	42	43.3
Yemen	12	12.4
Egypt	8	8.2
Other combinations	5	5.2
**Religious Identity**		
**Denomination**		
Christian	36	38.3
Muslim	58	61.7
**Cultural Identity**		
**PCP is MENA**		
Yes	59	64.1
No	33	35.9
**Importance of MENA to your identity (0–10, no-very) (mean, SD)**	8.0	2.8
**Health Behaviors**		
**Routine checkup**		
Within the last 3 years	87	95.6
3 or more years	4	4.4
Never	0	0.0
**Routine Pap test**		
Within the last 3 years	71	76.3
≥3 and < 5 years	4	4.3
More than 5 years ago	2	2.2
Never	16	17.2

PCP means primary care physician.

MENA means Middle East North Africa.

**Table 2:** Univariate association of religiosity on health care attitudes by MENA women^a^.

	I have avoided getting a routine physical because of religious/cultural issues^§^			I have avoided getting women’s health exams (OBGYN) because of religious/cultural issues^§^			I’m uncomfortable getting women’s health exams (OBGYN)^§^		
	*N* = 91			*N* = 92			*N* = 91		
	mean	SD	*p*-value	mean	SD	*p*-value	mean	SD	*p*-value
**Religiosity**									
**Denomination**			0.86			0.53			0.34
All others^Ɨ^	1.51	0.88		1.73	1.02		2.04	0.98	
Muslim	1.51	0.89		1.51	0.82		1.71	1.07	
**How often do you attend religious services?**			.029*			0.96			0.65
Never or less than once/yr	2.21	1.25		1.86	1.17		2.21	1.12	
Several times year	1.31	0.71		1.50	0.86		1.72	0.96	
About once a month	1.38	0.74		1.75	0.89		2.25	1.04	
2–3 times a month	1.56	0.73		1.56	0.73		2.00	1.00	
Greater than 3 times/mo	1.38	0.78		1.69	1.04		1.83	1.07	
**Religious Salience**									
**How Important is religion to you?**			0.27			0.73			0.31
Very important	1.55	0.94		1.64	0.94		1.93	1.07	
Fairly important	1.46	0.52		1.92	1.04		2.08	0.76	
Fairly unimportant	1.00	0.00		1.00	0.00		1.00	0.00	
Not at all important	NR			NR			NR		
**Religious meaning**									
**God/Allah put me in this life for a purpose**			0.83			0.42			0.75
Strongly Disagree	1.00	0.00		2.50	2.12		2.50	2.12	
Somewhat Disagree	1.00			1.00			1.00		
Somewhat Agree	1.68	0.99		1.69	0.97		1.76	0.88	
Strongly Agree	1.42	0.81		1.63	0.93		1.97	1.08	
**God/Allah has a specific plan for my life**			0.33			0.13			0.65
Strongly Disagree	1.00			4.00			4.00		
Somewhat Disagree	1.00	0.00		1.00	0.00		1.00	0.00	
Somewhat Agree	1.81	0.98		1.77	0.99		1.88	0.91	
Strongly Agree	1.37	0.81		1.56	0.88		1.89	1.08	
**God/Allah has a reason for everything that happens to me**			0.51			0.19			0.75
Strongly Disagree	1.00			4.00			4.00		
Somewhat Disagree	1.33	0.58		1.33	0.58		1.33	0.58	
Somewhat Agree	1.75	0.99		1.71	1.00		1.83	0.92	
Strongly Agree	1.42	0.84		1.58	0.89		1.89	1.06	

§ 1–4 scale (1 is strongly disagree 2 is somewhat disagree 3 is somewhat agree 4 is strongly agree).

Ɨ All others includes Chaldean, Catholic, Assyrian, Coptic, Jehovah Witness, Jewish, Baha’i, Druze or Alewi, Hindu, Buddhist, and Sikh.

a *p*-value based on univariate regression bootstrap techniques.

* *p* < 0.05.

**Table 3:** Association of sociocultural preferences for a physician by MENA women and MENA identity for health care attitudes.

Sociocultural preferences for physician	I have avoided getting a routine physical because of religious/cultural issues §			I have avoided getting women’s health exams (OBGYN) because of religious/cultural issues §			I’m uncomfortable getting women’s health exams (OBGYN) §		
	*N* = 91			*N* = 92			*N* = 91		
	mean	SD	*p*-value	mean	SD	*p*-value	mean	SD	*p*-value
**It is important for my healthcare provider to be of the same sex as me, that is, a female doctor for a female patient**			<.001***			0.014*			.002**
Strongly Disagree	1.22	0.66		1.38	0.83		1.56	1.01	
Somewhat Disagree	1.40	0.60		1.70	0.98		1.80	0.83	
Somewhat Agree	1.57	0.79		1.75	0.80		2.18	0.86	
Strongly Agree	2.45	1.37		2.18	1.33		2.45	1.37	
**It is important for my healthcare provider to be of the same religion as me**			<.001***			0.003**			.002**
Strongly Disagree	1.09	0.35		1.42	0.90		1.60	0.90	
Somewhat Disagree	1.74	0.86		1.70	0.78		2.11	0.97	
Somewhat Agree	2.18	0.98		2.27	0.90		2.55	0.93	
Strongly Agree	2.67	1.51		2.17	1.47		2.33	1.51	
**It is important for my healthcare provider to be of the same culture as me**			<.001***			0.017*			0.045*
Strongly Disagree	1.11	0.44		1.47	0.94		1.73	0.99	
Somewhat Disagree	1.67	0.70		1.67	0.76		1.92	0.93	
Somewhat Agree	2.18	1.13		2.12	0.99		2.41	1.06	
Strongly Agree	2.50	1.73		2.00	1.41		2.00	1.41	
**MENA Identity**									
**PCP MENA**			0.39			0.72			0.43
Yes	1.62	0.93		1.68	0.94		1.87	0.98	
No	1.36	0.78		1.58	1.00		1.91	1.10	
**How important is being MENA to your overall identity?**			0.78			0.40			0.13
None to moderate (0–8)	1.45	0.76		1.69	0.97		2.02	1.05	
Very important (9–10)	1.48	0.94		1.56	0.96		1.70	0.95	

Bold/red means statistically significant

* *p* < 0.05,

** *p* < 0.01,

*** *p* < 0.001

*p*-values based on bootstrap techniques

§ 1–4 scale (1 = strongly disagree 2 = somewhat disagree 3 = somewhat agree 4 = strongly agree)

PCP = primary care physician.

MENA = Middle East North Africa.

**Table 4:** Multiple regression model predicting the avoidance of routine physicals because of religious/cultural issues^§^.

Variable	Coefficient	Standard Error (Bootstrap)	95% CI (Bias-Corrected)	*p*-value (Bootstrap)
Education^a^	−0.083	0.04	(−.16, −0.01)	0.03
Religious Frequency^b^	−0.062	0.04	(−.13, 0.01)	0.08
Same Sex^c^	0.126	0.07	(−.013, 0.26)	0.07
Same Religion^d^	0.40	0.10	0.21, 0.59)	< 0.001
Same Culture^e^	0.212	0.09	(0.03, 0.40)	0.03

§ 1–4 scale (1 = strongly disagree 2 = somewhat disagree 3 = somewhat agree 4 = strongly agree).

a Education ranked from high school or less- some college-college- post-college.

b Religious frequency ranked from: never or less than once/year – several times/year – about once a month- 2–3 times a month – greater than 3 times/month.

c Same sex means: **It is important for my healthcare provider to be of the same sex as me, that is, a female doctor for a female patient.** And is ranked from strongly disagree-somewhat disagree-somewhat agree- strongly agree.

d Same religion means: **It is important for my healthcare provider to be of the same religion as me.** And is ranked from strongly disagree-somewhat disagree-somewhat agree- strongly agree.

e Same culture means: **It is important for my healthcare provider to be of the same culture as me.** And is ranked from strongly disagree-somewhat disagree-somewhat agree- strongly agree.

**Table 5:** Multiple regression model predicting the avoidance of women’s health exams because of religious/cultural issues^§^.

Variable	Coefficient	Standard Error (Bootstrap)	95% CI (Bias-Corrected)	*p*-value (Bootstrap)
Same Sex^a^	0.138	0.11	(−0.05, 0.32)	0.21
Same Religion^b^	0.270	0.13	(0.04, 0.57)	0.03
Same Culture^c^	0.062	0.12	(−0.16, 0.29)	0.62

§ 1–4 scale (1 = strongly disagree 2 = somewhat disagree 3 = somewhat agree 4 = strongly agree).

a Same sex means: **It is important for my healthcare provider to be of the same sex as me, that is, a female doctor for a female patient.** And is ranked from strongly disagree-somewhat disagree-somewhat agree- strongly agree.

b Same religion means: **It is important for my healthcare provider to be of the same religion as me.** And is ranked from strongly disagree-somewhat disagree-somewhat agree- strongly agree.

c Same culture means: **It is important for my healthcare provider to be of the same culture as me.** And is ranked from strongly disagree-somewhat disagree-somewhat agree- strongly agree.

**Table 6:** Multiple regression model predicting being uncomfortable getting women’s health exams^§^.

Variable	Coefficient	Standard Error	95% CI (Bias-Corrected)	*p*-value
Same Sex^a^	0.232	0.11	(0.01, 0.44)	0.043
Same Religion^b^	0.273	0.13	(0.04, 0.54)	0.037

§ 1–4 scale (1 = strongly disagree 2 = somewhat disagree 3 = somewhat agree 4 = strongly agree).

a Same sex means: **It is important for my healthcare provider to be of the same sex as me, that is, a female doctor for a female patient.** And is ranked from strongly disagree-somewhat disagree-somewhat agree-strongly agree.

b Same religion means: **It is important for my healthcare provider to be of the same religion as me.** And is ranked from strongly disagree-somewhat disagree-somewhat agree-strongly agree.

## Data Availability

De-identified data and data dictionaries are available at: https://www.openicpsr.org/openicpsr/project/148081/version/V1/view

## Research in context

### Evidence before this study

Arab American ethnicity was not a priority identifier in research in the United States (US) prior to September 11, 2001 when the associated Muslim religion was seen as dissonant with US culture. While the majority of Arab Americans immigrating were of Christian faiths, the Muslim practices challenged healthcare systems to provide healthcare. The first reports describing the divergences were qualitative interviews in 2004 indicating the importance of the concordance of a female physician ideally of the Muslim faith to provide women’s health exams. This was driven by the Muslim tenets of modesty, virginity, and male dominance of the family. Over the next two decades, women’s health exams for the Middle East North African (MENA) population in the US were reported through national surveillance surveys, state-specific cancer surveys, and site-specific practices, with implicit, but undocumented, understanding that women’s health exams were conducted by female physicians preferably of Arab descent. These studies show women’s health exams did occur, but not the reasoning or priority for attendance.

### Added value of this study

Our work details the changes in the religious and cultural aspects that influence Arab American women’s perspectives on healthcare in the US. MENA women no longer necessarily prefer a physician of the same sex, religion, or culture. Instead, MENA’s women’s avoidance of health exams appears to occur because of the physician’s perceived inability to separate their religion/culture from their practice of medicine.

### Implications of all the available evidence

The religion and culture of MENA women remain one of the most significant reasons for avoiding routine and women’s healthcare. While there has been a concerted effort to increase the cultural competency of physicians who are unlike the religion and culture of MENA women over the past decade, future work must address the professionalism of separating the physician’s religion/culture from the practice of evidence-based medicine so that MENA woman have a safe space for healthcare.
