# Supplementary material for: Concordant physician-patient characteristics lose importance for Arab American women and their healthcare- cross-sectional study
Source: Lancet Reg Health Am. 2022 Mar 3;10:100225. doi: 10.1016/j.lana.2022.100225 (PMC9236211; doi:10.1016/j.lana.2022.100225)
Supplement: Supplementary file 1 [file mmc1.docx]

**Supplemental Table 1**. Avoidance of Health Behaviors by Covariates

|  | I have avoided getting a **routine physical** because of **religious/cultural issues**^§^ | | | I have avoided getting **women's health exams** (OBGYN) because of **religious/cultural issues**^§^ | | | I’m uncomfortable getting **women's health exams** (OBGYN)^§^ | | |
| --- | --- | --- | --- | --- | --- | --- | --- | --- | --- |
|  | N=91 | | | N=92 | | | N=91 | | |
|  | mean | SD | p-value | mean | SD | p-value | mean | SD | p-value |
| **Covariates** |  |  |  |  |  |  |  |  |  |
| **Marital Status** |  |  |  |  |  |  |  |  |  |
| Married | 1.58 | 0.94 |  | 1.64 | 0.95 |  | 1.96 | 1.05 |  |
| Single | 1.30 | 0.57 |  | 1.70 | 0.92 |  | 1.75 | 0.91 |  |
| **Education** |  |  | <0.05 |  |  |  |  |  |  |
| High School or less | 1.83 | 0.92 |  | 1.78 | 0.87 |  | 2.03 | 0.89 |  |
| Some college | 1.38 | 0.62 |  | 1.50 | 0.63 |  | 1.88 | 0.96 |  |
| College | 1.23 | 0.76 |  | 1.61 | 1.12 |  | 1.84 | 1.19 |  |
| Post college | 1.56 | 1.13 |  | 1.56 | 1.13 |  | 1.78 | 1.09 |  |
| **Income** |  |  |  |  |  |  |  |  |  |
| <$10K | 1.83 | 0.94 |  | 1.62 | 0.65 |  | 2.08 | 1.00 |  |
| $10-$49999 | 1.53 | 0.81 |  | 1.87 | 0.97 |  | 2.04 | 0.98 |  |
| $50-$99999 | 1.21 | 0.71 |  | 1.32 | 0.95 |  | 1.58 | 1.02 |  |
| >$100,000 | 1.55 | 1.04 |  | 1.64 | 1.12 |  | 2.00 | 1.26 |  |
| **Occupation** |  |  |  |  |  |  |  |  |  |
| Employed | 1.48 | 0.98 |  | 1.66 | 1.06 |  | 1.77 | 1.05 |  |
| Unemployed | 1.65 | 0.70 |  | 1.83 | 0.86 |  | 2.06 | 0.75 |  |
| Homemaker | 1.44 | 0.78 |  | 1.50 | 0.79 |  | 2.00 | 1.08 |  |
| Disabled | 1.00 | 0.00 |  | 1.00 | 0.00 |  | 1.00 | 0.00 |  |
| **Insurance** |  |  |  |  |  |  |  |  |  |
| Private | 1.32 | 0.86 |  | 1.57 | 1.03 |  | 1.96 | 1.20 |  |
| Federal | 1.59 | 0.77 |  | 1.64 | 0.91 |  | 1.90 | 0.89 |  |
| None |  |  |  |  |  |  |  |  |  |
| **Mediators** |  |  |  |  |  |  |  |  |  |
| **Parent's Country of Origin** |  |  |  |  |  |  |  |  |  |
| Lebanon | 1.54 | 1.04 |  | 1.66 | 1.01 |  | 1.89 | 1.07 |  |
| Iraq | 1.59 | 0.89 |  | 1.59 | 0.89 |  | 1.85 | 1.09 |  |
| Yemen | 1.45 | 0.52 |  | 2.09 | 1.14 |  | 1.91 | 0.83 |  |
| Egypt | 1.50 | 0.84 |  | 1.67 | 0.82 |  | 2.17 | 0.75 |  |
| Other combinations | 1.00 | 0.00 |  | 1.20 | 0.45 |  | 2.20 | 1.10 |  |
| **Born in US** |  |  |  |  |  |  |  |  |  |
| Yes | 1.33 | 0.90 |  | 1.47 | 0.99 |  | 2.00 | 1.20 |  |
| No | 1.55 | 0.87 |  | 1.69 | 0.94 |  | 1.89 | 0.99 |  |
| **Length of time in US** |  |  |  |  |  |  |  |  |  |
| 10 years or less | 1.67 | 0.69 |  | 1.83 | 0.71 |  | 2.11 | 0.68 |  |
| More than 10 years | 1.47 | 0.92 |  | 1.58 | 0.98 |  | 1.80 | 1.10 |  |
| **Healthcare Attendance** |  |  |  |  |  |  |  |  |  |
| **Routine checkup** |  |  |  |  |  |  |  |  |  |
| Within the last 3 years | 1.14 | 0.35 |  | 1.19 | 0.39 |  | 1.27 | 0.45 |  |
| 3 or more years | 1.00 | 0.00 |  | 1.00 | 0.00 |  | 1.50 | 0.58 |  |
| **Routine Pap test** |  |  |  |  |  |  |  |  |  |
| Within the last 3 years | 1.14 | 0.35 |  | 1.19 | 0.39 |  | 1.28 | 0.45 |  |
| ≥3 and < 5 years | 1.00 | 0.00 |  | 1.25 | 0.50 |  | 1.25 | 0.50 |  |
| More than 5 years ago | 1.50 | 0.71 |  | 1.00 | 0.00 |  | 1.00 | 0.00 |  |
| Never | 1.13 | 0.35 |  | 1.13 | 0.35 |  | 1.33 | 0.49 |  |

§ 1-4 scale (1 = strongly disagree 2 = somewhat disagree 3 = somewhat agree 4 = strongly agree)
